# Mental Health Help-Seeking in Parents and Trajectories of Depressive and Anxiety Symptoms: Lessons Learned From the Ontario Parent Survey During the COVID-19 Pandemic

**DOI:** 10.3389/fpsyg.2022.884591

**Published:** 2022-06-16

**Authors:** Xutong Zhang, Marc Jambon, Tracie O. Afifi, Leslie Atkinson, Teresa Bennett, Eric Duku, Laura Duncan, Divya Joshi, Melissa Kimber, Harriet L. MacMillan, Andrea Gonzalez

**Affiliations:** ^1^Department of Psychiatry and Behavioral Neurosciences, McMaster University, Hamilton, ON, Canada; ^2^Offord Centre for Child Studies, Hamilton, ON, Canada; ^3^Department of Community Health Sciences, University of Manitoba, Winnipeg, MB, Canada; ^4^Department of Psychology, Toronto Metropolitan University, Toronto, ON, Canada

**Keywords:** COVID-19, depression, anxiety, parent, mental health, help-seeking

## Abstract

Tracking parents’ mental health symptoms and understanding barriers to seeking professional help are critical for determining policies and services to support families’ well-being. The COVID-19 pandemic has posed enormous challenges to parents’ mental health and the access to professional help, and there are important public health lessons that must be learned from the past 2 years’ experiences to inform future mental health responses to social- and family-level stressful events. This study examines the trajectories of parents’ depressive and anxiety symptoms over a year during the pandemic as related to their mental health help-seeking. Data were collected from a sample of parents residing in Ontario, Canada at baseline (May–June, 2020; Wave 1) and again 1 year later (Wave 2; referred to as W1 and W2 below). Parents (*n* = 2,439; *M*_age_ = 39.47, *SD* = 6.65; 95.0% females) reported their depressive and anxiety symptoms at both waves. Mental health help-seeking, including self-reported contact with professional help and perceived unmet mental health needs, was measured at W2. Parents were classified into four groups by mental health help-seeking. *Inconsistent seekers* and *non-seeking needers*, both reporting perceived unmet needs for professional help, showed greater increases in depressive and anxiety symptoms, whereas parents with *no need* or *needs met* showed smaller increases in depressive symptoms and decreases in anxiety symptoms. Belief in self-reliance and time constraints were the leading reasons for not seeking help. These findings suggest that over a year into the pandemic, parents with perceived unmet mental health needs were at greater risk for worsening depressive and anxiety symptoms. Recognizing the demands for mental health services when families experience chronic stressors and targeting the identified barriers may promote family well-being during and beyond this pandemic.

## Introduction

With effects of the virus on morbidity and mortality and the prolonged disruption to daily lives, the COVID-19 pandemic has posed enormous challenges to people’s mental health (Pfefferbaum and North, 2020; Vindegaard and Benros, 2020). Parents, especially mothers of school-age or younger children, showed particularly high rates of psychological distress early in the pandemic (Pierce et al., 2020; Gadermann et al., 2021; Zamarro and Prados, 2021). The mental toll on parents is not surprising given the increased burden of childcare during school closures, disruptions to family routines and income, and concerns about children’s well-being (Racine et al., 2021). Parents’ mental health problems impact their own well-being as well as family functioning and children’s development (Russell et al., 2020). Therefore, it is critical to track parents’ mental health symptoms to inform how to support families’ well-being during this global health crisis, the recovery from its profound impact, as well as future stressful situations for families.

Since the pandemic started, recurring surges of infections, including the most recent spread of Omicron variant, have led to chronic stressors. Related mental health research is thus shifting from examining the initial acute impact to understanding how people adapt to longer-term adversity, aiming to provide guidance for mental health responses to future social- and family-level stressors (Stark et al., 2020). Some studies indicate that psychological distress in the general population of several western countries (e.g., United States and Germany) decreased following a peak in April 2020 (Bendau et al., 2021; Daly and Robinson, 2021). However, further evidence shows that such trends were due to a decline in anxiety as people learned more about the virus and vaccines became available, whereas depressive symptoms persisted (Zamarro and Prados, 2021). It is unclear whether similar patterns exist among parents who face unique challenges during the pandemic. More importantly, there is a lack of evidence on the situation around parents’ mental health help-seeking and its association with trajectories of mental health symptoms.

Health and mental health professionals (e.g., family doctors and psychiatrists) constitute an important source of service and support to address individuals’ mental health needs. However, a variety of factors, such as stigma, belief in self-reliance, and lack of access, may prevent individuals from seeking help (Gulliver et al., 2010). The pandemic has presented additional barriers such as discomfort with in-person visits and technological barriers to virtual services, and parents may also face time constraints due to increased childcare responsibilities. These factors can make it difficult for parents to consistently seek professional help when experiencing mental health challenges, contributing to unmet needs (Cameron et al., 2020). To fully understand parents’ mental health help-seeking, research must consider it as a multidimensional construct, defined not only by individuals’ contacts with professionals due to mental health concerns, but also by their perceived unmet needs (i.e., needed but did not seek help).

The current study aims to understand trajectories in mental health symptoms as related to mental health help-seeking during the pandemic in a sample of parents residing in Ontario—the most populated province in Canada. By the fall of 2021, Ontario had experienced three waves of COVID-19 case surges and public health restrictions (March–July, 2020; November, 2020–February, 2021; and March–May, 2021). In the course of 16 months from March, 2020 to August, 2021, in-person schooling was closed for more than 20 weeks (Gallagher-Mackay et al., 2021). Upon re-opening, children are required to stay at home in the case of COVID-related situations (e.g., showing COVID symptoms, exposed to classmates who test positive, and school outbreak), thereby placing continued demands on parents. Using longitudinal data from the Ontario Parent Survey (OPS), this study examines whether and how the trajectories of parents’ mental health symptoms differ by their mental health help-seeking, classified based on contacts with health or mental health professionals and perceived unmet mental health needs. We hypothesize that parents with no unmet mental health needs would be at lower risk for worsening depressive or anxiety symptoms compared to those with inconsistent help-seeking (i.e., had contacts with professional help but still reported unmet needs); furthermore, parents with unmet needs who reported no contact with professional help at all would be at the greatest risk for worsening symptoms.

## Materials and Methods

### Participants

This study used data from the OPS, a project examining families’ experiences during the COVID-19 pandemic in Ontario, Canada. From May 5 to June 19, 2020 (W1; the first provincial stay-at-home order), a convenience sample of parents with children aged 0–17 years was recruited through crowdsourcing techniques, including advertisements online through social media platforms and email announcements through public health units, Ontario EarlyON Centers, school boards, and municipal, community, and professional organizations (e.g., Ontario Nurses Association, across Ontario). Among 6,686 parents who participated in W1, 3,475 consented to be contacted for follow-up and were invited to participate in W2. W2 data collection occurred between May 4 and July 1, 2021, and largely overlapped with the third provincial stay-at-home order (April 8–June 2, 2021; schools remained closed until September). In both waves, electronic informed consent was obtained. Participants completed the survey online in English or French. The study was approved by the McMaster University Hamilton Integrated Research Ethics Board.

Of the parents who consented to follow-up, 70.2% responded to the W2 survey and were included in this study (*n* = 2,439; see Supplementary Table 1 in Supplementary Material for comparisons between the final sample and those who did not consent or respond to W2). Based on W1 data, the final sample (*M*_age_ = 39.47, *SD* = 6.65, ranging from 21 to 73 years) consisted of 95.0% females, 4.8% males, and 0.2% genderqueer/non-binary individuals. Most parents identified as North American or European (89.1%) with the rest identifying as East/Southeast Asian, First Nations, South Asian, Metis, Latin/Central/South American, Caribbean, Middle Eastern, African, Oceanic, or other ethnicity. The majority of parents were married or living with a common-law partner (86.0%), and over half held a bachelor’s degree or above (59.3%). Most parents worked full-time (64.0%) or part-time (12.4%). Parents reported having 1–12 children (*M*_# of children_ = 1.97, *SD* = 0.94), and 51.8% had at least one child under 5 years.

### Measures of Mental Health Help-Seeking

At W2, parents reported their contacts with health or mental health professionals (i.e., whether they had seen or talked to any healthcare provider because of mental health concerns) and perceived unmet mental health needs (i.e., whether there were times they needed but did not seek professional help) since the beginning of the pandemic. They also indicated the type(s) of providers they had contact with and the reasons for not seeking help (see Supplementary Table 2 in Supplementary Material; parents were asked to mark all that applied). For our study aim, parents were categorized into four help-seeking groups labeled as (1) *no need*—had no contact with professional help and reported no unmet needs (*n* = 674; 31.5%), (2) *needs met*—had contacts with professional help and reported no unmet needs (*n* = 552; 25.8%), (3) *inconsistent seekers*—had contacts but also reported unmet needs (*n* = 487; 22.8%), and (4) *non-seeking needers*—had no contact with professional help and reported unmet needs (*n* = 427; 20.0%). See Table 1 for group characteristics.

**Table 1:** Sample characteristics by help-seeking group.^a^

	No need (*n* = 674)	Needs met (*n* = 552)	Inconsistent seekers (*n* = 487)	Non-seeking needers (*n* = 427)	Group comparison
Age in years, mean (*SD*)	40.60 (6.62)	39.76 (6.56)	38.56 (6.84)	39.15 (6.31)	*F* (3, 2,136) = 9.89, *p* < 0.001
Female, No. (%)	627 (93.0)	526 (95.3)	466 (95.7)	408 (95.6)	*χ*^2^(3) = 5.53, *p* = 0.14
Ethnic minority,^b^ No. (%)	57 (8.5)	67 (12.1)	54 (11.1)	29 (6.8)	*χ*^2^(3) = 10.20, *p* = 0.02
Married, No. (%)	600 (89.0)	457 (82.8)	409 (84.0)	383 (89.7)	*χ*^2^(3) = 15.18, *p* = 0.002
College degree, No. (%)	427 (63.4)	311 (56.3)	281 (57.7)	275 (64.4)	*χ*^2^(3) = 9.73, *p* = 0.02
Working full-time, No. (%)	440 (65.3)	356 (64.5)	299 (61.4)	286 (67.0)	*χ*^2^(3) = 3.21, *p* = 0.36
Have child(ren) <5 years, No. (%)	305 (45.3)	277 (50.2)	276 (56.7)	227 (53.2)	*χ*^2^(3) = 16.05, *p* = 0.001
COVID financial impact,^c^ mean (*SD*)	0.54 (0.77)	0.78 (0.94)	0.91 (0.96)	0.65 (0.87)	*F* (3, 1,980) = 17.26, *p* < 0.001
Depressive symptoms					
W1: CESD ≥ 10, No. (%)	228 (34.1)	334 (60.6)	392 (80.7)	274 (64.5)	χ^2^(3) = 266.64, *p* < 0.001
W2: CESD ≥ 10, No. (%)	295 (44.2)	374 (67.8)	425 (87.8)	336 (78.9)	χ^2^(3) = 280.26, *p* < 0.001
Change^d^ from W1 to W2	1.14 (4.99)	1.36 (6.66)	2.37 (6.24)	3.27 (5.56)	*F* (3, 2,118) = 14.06, *p* < 0.001
Anxiety symptoms					
W1: GAD ≥ 10, No. (%)	142 (21.1)	208 (37.9)	236 (48.8)	141 (33.1)	χ^2^(3) = 100.26, *p* < 0.001
W2: GAD ≥ 10, No. (%)	82 (12.2)	175 (31.7)	297 (61.1)	193 (45.2)	χ^2^(3) = 321.52, *p* < 0.001
Change^d^ from W1 to W2	−1.52 (6.46)	−0.96 (6.69)	1.10 (6.07)	1.41 (5.35)	*F* (3, 2,124) = 29.24, *p* < 0.001

*CESD, the sum score on CES-D-10; GAD, the sum score on GAD-7*.

a *This table characterizes 2,140 parents who responded to the help-seeking questions. The percentages were calculated using the non-missing group size for each variable as denominator*.

b *Ethnicity status other than North American and European was coded as ethnic minority*.

c *The impact of COVID on family financial obligations*.

d *Change in sum scores from W1 to W2*.

### Measures of Depressive and Anxiety Symptoms

Depressive symptoms were measured at both waves using the 10-item Center for Epidemiologic Studies Depression Scale (CES-D-10; Andresen et al., 1994). Parents rated how often they experienced each symptom over the past week on a 0 (rarely or never) to 3 (all the time) Likert scale. This scale had excellent internal consistency in the current sample (Cronbach’s *α* was 0.87 at W1 and 0.88 at W2). A sum score was calculated at each wave (possible range = 0–30).

Anxiety symptoms were measured at both waves using the seven-item Generalized Anxiety Disorder Scale (GAD-7; Spitzer et al., 2006). Parents rated how often they experienced each symptom over the past 2 weeks on a 0 (not at all) to 3 (nearly everyday) Likert scale. The internal consistency of the scale was excellent in the current sample (Cronbach’s *α* was 0.91 at W1 and 0.90 at W2). A sum score was calculated at each wave (possible range = 0–21).

### Covariates

Based on previous studies of general or pandemic-specific risk factors for psychological distress (Pierce et al., 2020; Xiong et al., 2020), the following W1 variables were included as covariates: parents’ self-reported age, gender, ethnicity, marital status, education, employment, whether they have child(ren) under 5 years, and the impact of COVID on family financial obligations (referred to as “COVID financial impact” below). COVID financial impact was measured by a single item “the impact of COVID-19 on your ability to meet financial obligations or essential needs, such as rent or mortgage payments, utilities, and groceries,” rated on a 0 (no impact) to 3 (major impact) ordinal scale. Categorical covariates were dichotomously coded into 0 and 1 (see Table 1).

### Statistical Analysis

The analyses examined whether trajectories of parents’ depressive and anxiety symptoms (i.e., changes in sum scores on CES-D-10 and GAD-7 from W1 to W2) differed by help-seeking groups. Descriptive analyses on depressive and anxiety symptoms were conducted first, and paired-sample *t*-tests examined how symptoms changed between waves across the sample. Then, the hypothesis was tested using two structural equation models (see Supplementary Figure 1 in Supplementary Material). W1 level of symptoms and a latent change score representing the change between waves were regressed on covariates and help-seeking groups. All covariates were centered around sample means so that intercepts are more interpretable. Help-seeking groups were recoded into three variables based on a dummy coding scheme, examining how three of the groups (*needs met*, *inconsistent seekers*, and *non-seeking needers*) compared to the reference group (*no need*).

The models were fit to the data using the *lavaan* package (version 0.6-9; Rosseel, 2012) in R Core Team (2020) with full-information maximum likelihood estimation. In the final sample, 326 parents (13.4%) had incomplete data among five key variables (help-seeking, depressive, and anxiety symptoms at two waves; > 99% of the 326 parents were missing 3 or less variables). Having incomplete data was associated with several covariates, including younger parental age, ethnic minority status, not having a college degree, having child(ren) under 5 years of age, and greater COVID financial impact, but not with W1 depressive or anxiety symptoms. Incomplete data were treated as missing-at-random, with all covariates associated with the missingness included in the analysis. Model fit was evaluated based on comparative fit index (CFIcomparative fit index; >0.90), Tucker–Lewis index (TLI; >0.90), Root Mean Square Error of Approximation (RMSEA; <0.08), and Standardized Root Mean Square Residual (SRMR; <0.08; Bentler, 2007). Absolute fit index (χ^2^) was not used here as it is over-sensitive to model-data discrepancy with large sample sizes in multivariate models (Fan et al., 1999). Statistical significance was evaluated with two-tailed tests at *α* = 0.05.

## Results

Based on the widely used cutoff for CES-D-10 (i.e., a sum score ≥ 10; Mohebbi et al., 2018; Kiviruusu et al., 2020), 58.1 and 67.4% of parents in this sample reported clinically significant depressive symptoms at W1 (*M*_W1_ = 11.52, *SD* = 6.40) and W2 (*M*_W2_ = 13.33, *SD* = 6.86). Using a cutoff score ≥ 10 for GAD-7 (Plummer et al., 2016), 34.8 and 35.2% of parents reported moderate to severe anxiety symptoms at W1 (*M*_W1_ = 8.05, *SD* = 5.76) and W2 (*M*_W2_ = 7.84, *SD* = 5.55). Paired-sample *t*-tests showed that, on average, parents’ depressive symptoms increased significantly from W1 to W2, *t*(2,191) = 14.97, *p* < 0.001, whereas anxiety symptoms did not change significantly, *t*(2,193) = −1.55, *p* = 0.12.

### Trajectories of Parental Mental Health Symptoms by Help-Seeking Groups

The two structural equation models showed good fit (depression model: CFI = 0.98, TLI = 0.95, RMSEA = 0.03, and SRMR = 0.03; anxiety model: CFI = 0.97, TLI = 0.93, RMSEA = 0.03, and SRMR = 0.03). Estimates of regression paths are presented in Tables 2, 3. Overall, results suggested that the trajectories of parental mental health symptoms differed by help-seeking groups.

**Table 2:** Parameter estimates of regression paths in the depression model.

Predictor	Depression model	
	*b* (*SE*)	*β*, 95% CI
*Predicting W1 level*		
Age in years	−0.04 (0.02)	−0.04, [−0.09, 0.01]
Female	0.06 (0.56)	0.002, [−0.03, 0.04]
Ethnic minority	0.17 (0.42)	0.008, [−0.03, 0.05]
Married	−1.19^**^ (0.36)	−0.06, [−0.10, −0.03]
College degree	−0.20 (0.25)	−0.02, [−0.05, 0.02]
Working full-time	−0.52^*^ (0.26)	−0.04, [−0.08, −0.001]
Have child <5 years	0.34 (0.29)	0.03, [−0.02, 0.07]
COVID financial impact	1.00^***^ (0.15)	0.14, [0.10, 0.18]
Group: Needs met	3.36^***^ (0.34)	0.23, [0.19, 0.27]
Group: Inconsistent seekers	6.37^***^ (0.34)	0.42, [0.38, 0.46]
Group: Non-seeking needers	3.76^***^ (0.33)	0.24, [0.19, 0.28]
*Predicting change between waves*
Age in years	0.03 (0.02)	0.03, [−0.02, 0.08]
Female	0.40 (0.51)	0.01, [−0.02, 0.05]
Ethnic minority	−0.57 (0.43)	−0.03, [−0.07, 0.01]
Married	0.86^*^ (0.37)	0.05, [0.01, 0.09]
College degree	−0.15 (0.27)	−0.01, [−0.06, 0.03]
Working full-time	0.10 (0.27)	0.008, [−0.04, 0.05]
Have child <5 years	0.01 (0.30)	0.001, [−0.05, 0.05]
COVID financial impact	0.01 (0.16)	0.001, [−0.05, 0.05]
Group: Needs met	0.33 (0.35)	0.02, [−0.03, 0.07]
Group: Inconsistent seekers	1.34^***^ (0.35)	0.09, [0.05, 0.14]
Group: Non-seeking needers	2.16^***^ (0.34)	0.15, [0.10, 0.19]

**b* (*SE*), unstandardized coefficient (standard error); *β*, 95% CI, standardized coefficient, 95% confidence interval. Effects of the three groups compare them to the reference “no need” group*.

* **p* < 0.05;*

** *p* *< 0.01;*

*** *p < 0.001*.

**Table 3:** Parameter estimates of regression paths in the anxiety model.

Predictor	Anxiety model	
	*b* (*SE*)	*β*, 95% CI
*Predicting W1 level*		
Age in years	−0.04^*^ (0.02)	−0.05, [−0.10, −0.001]
Female	−0.54 (0.58)	−0.02, [−0.06, 0.02]
Ethnic minority	0.53 (0.39)	0.03, [−0.01, 0.07]
Married	0.13 (0.34)	0.008, [−0.03, 0.05]
College degree	−0.32 (0.24)	−0.03, [−0.07, 0.01]
Working full-time	0.08 (0.24)	0.006, [−0.03, 0.05]
Have child <5 years	−0.15 (0.28)	−0.01, [−0.06, 0.04]
COVID financial impact	0.70^***^ (0.14)	0.11, [0.07, 0.15]
Group: Needs met	2.13^***^ (0.34)	0.16, [0.11, 0.21]
Group: Inconsistent seekers	3.47^***^ (0.33)	0.25, [0.20, 0.30]
Group: Non-seeking needers	1.68^***^ (0.33)	0.12, [0.07, 0.16]
*Predicting change between waves*
Age in years	−0.02 (0.02)	−0.02, [−0.07, 0.03]
Female	1.11 (0.62)	0.04, [−0.003, 0.08]
Ethnic minority	−0.81 (0.48)	−0.04, [−0.09, 0.01]
Married	0.15 (0.36)	0.008, [−0.03, 0.05]
College degree	−0.03 (0.28)	−0.003, [−0.04, 0.04]
Working full-time	−0.41 (0.28)	−0.03, [−0.07, 0.01]
Have child <5 years	0.50 (0.32)	0.04, [−0.01, 0.09]
COVID financial impact	−0.04 (0.16)	−0.006, [−0.05, 0.04]
Group: Needs met	0.57 (0.38)	0.04, [−0.01, 0.09]
Group: Inconsistent seekers	2.59^***^ (0.38)	0.17, [0.12, 0.22]
Group: Non-seeking needers	2.87^***^ (0.36)	0.18, [0.14, 0.22]

**b* (*SE*), unstandardized coefficient (standard error); *β*, 95% CI, standardized coefficient, 95% confidence interval. Effects of the three groups compare them to the reference “*no need*” group*.

* *p* *< 0.05 and*

*** *p < 0.001*.

**Figure 1:**
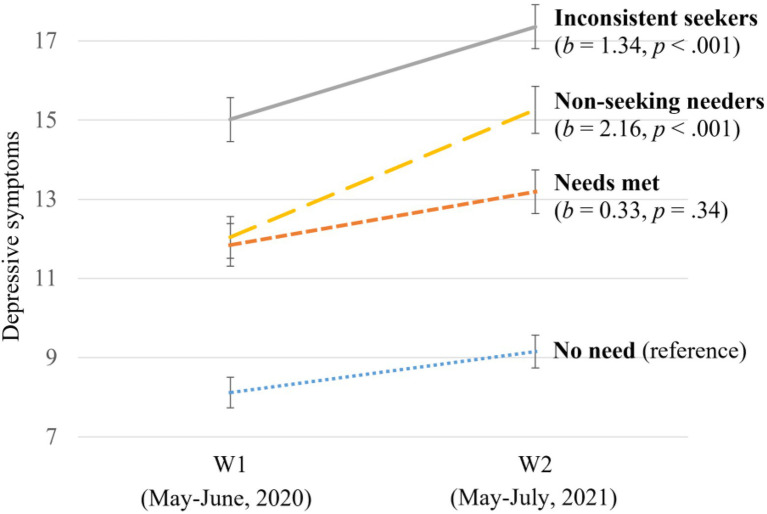
Changes in parents’ depressive symptoms between waves by help-seeking group. The coefficient (*b*s) and corresponding *p*-values indicate how the changes in depressive symptoms in the three groups differed from the reference group after controlling for covariates. The error bars indicate 95% CIs of symptom levels.

Trajectories of parental depressive symptoms in each group are illustrated in Figure 1. In the *no need* group, the average level of depressive symptoms at W1 was μ_depW1_ = 8.41, *SE* = 0.20, *p* < 0.001, and there was a significant increase from W1 to W2 (μ_Δdep_ = 1.05, *SE* = 0.20, *p* < 0.001). Compared to parents with *no need*, the *needs met* group, although reporting higher levels of depressive symptoms at W1 (*b* = 3.36, *p* < 0.001), did not show steeper increases in symptoms between waves (*b* = 0.33, *p* = 0.34). In contrast, *inconsistent seekers* and *non-seeking needers* both reported higher levels of depressive symptoms at W1 (*inconsistent seekers*: *b* = 6.37, *p* < 0.001; *non-seeking needers*: *b* = 3.76, *p* < 0.001) and steeper increases between waves (*inconsistent seekers*: *b* = 1.34, *p* < 0.001; *non-seeking needers*: *b* = 2.16, *p* < 0.001) compared to parents with no need. *Post hoc* comparisons were conducted by re-running the model with reference group switched; results suggested *inconsistent seekers* and *non-seeking needers* showed comparable increases in depressive symptoms between waves, both significantly steeper than the *needs met* group.

**Figure 2:**
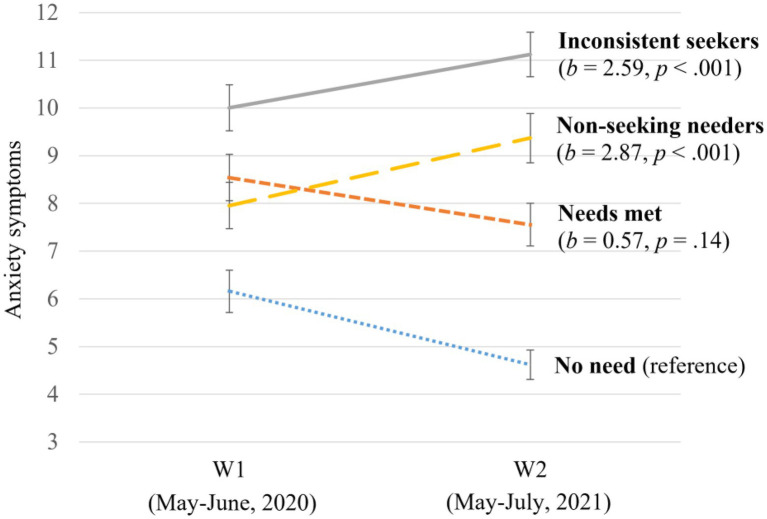
Changes in parents’ anxiety symptoms between waves by help-seeking group. The coefficient (*b*s) and corresponding *p* values indicate how the changes in anxiety symptoms in the three groups differed from the reference group after controlling for covariates. The error bars indicate 95% CIs of symptom levels.

Figure 2 illustrates trajectories in parental anxiety symptoms by help-seeking groups. The average level of anxiety symptoms in the *no need* group at W1 was μ_anxW1_ = 6.36, *SE* = 0.22, *p* < 0.001, which decreased significantly from W1 to W2 (μ_Δanx_ = −1.50, *SE* = 0.25, *p* < 0.001). Parents with *needs met* reported higher levels of anxiety symptoms at W1 than parents with *no need* (*b* = 2.13, *p* < 0.001), but also showed similar decreases in anxiety symptoms (*b* = 0.57, *p* = 0.14). Compared to parents with *no need*, *inconsistent seekers*, and *non-seeking needers* reported higher levels of anxiety symptoms at W1 (*inconsistent seekers*: *b* = 3.47, *p* < 0.001; *non-seeking needers*: *b* = 1.68, *p* < 0.001) and increases between waves (*inconsistent seekers*: *b* = 2.59, *p* < 0.001; *non-seeking needers*: *b* = 2.87, *p* < 0.001). *Post hoc* group comparisons again suggested that *inconsistent seekers* and *non-seeking needers* showed comparable increases in anxiety between waves, which were significantly different from trajectories in the *needs met* group.

In summary, the hypothesis was partially supported. Consistent with hypothesis, the two groups with unmet mental health needs showed greater increases in depressive and anxiety symptoms. However, the trajectories of symptoms among *inconsistent seekers* were comparable with *non-seeking needers*, who had no contact with health or mental health professionals and were predicted to be at the greatest risk for worsening symptoms.

### Mental Health Help-Seeking and Barriers

Approximately half of the sample (48.6%) reported having contacts with professionals because of mental health concerns since the beginning of the pandemic. Among these parents, the majority saw or talked to family doctors/general practitioners (77%), and smaller proportions reported having contacts with other types of professionals, including social workers (24%), psychologists (19%), psychiatrists (13%), nurses (9%), and other types of counselors (22%). Parents were asked to select all that applied. Among parents who had contacts with family doctors/general practitioners about mental health concerns (which may be the first point of access to the service system), over half (55.4%) also reported having contacts with other type(s) of professionals. Meanwhile, 42.7% of the sample reported perceived unmet needs. Several reasons for not seeking services were reported including, belief in self-reliance (i.e., thought they could manage it by themselves; 56%), followed by being too busy (46%) or scheduling difficulties (39%). Less than a quarter of parents indicated that the potential cost of services (22%) or reluctance around telehealth (20%) prevented them from seeking help. Only small proportions (≤10%) reported logistical reasons (e.g., hard to get to the location), lack of access (e.g., did not know where to get help; long waiting period), lack of faith in professional help, or worry of stigma. Among parents with perceived unmet needs, 37.0% reported one type of barrier, whereas the majority selected multiple barriers.

## Discussion

Using longitudinal data from the OPS, this study examined trajectories of parents’ depressive and anxiety symptoms in association with mental health help-seeking during the COVID-19 pandemic. Previous studies have indicated a substantial increase in psychological distress among Canadian parents when the pandemic was first declared compared to pre-pandemic (Racine et al., 2021; Shields et al., 2021). Our findings suggest that 1 year into the pandemic, although anxiety decreased among some parents (those reporting no perceived unmet mental health needs), depressive symptoms increased regardless of parents’ mental health help-seeking. Decreasing anxiety but persistent depressive symptoms during the pandemic was also found in a representative sample of U.S. adults (Zamarro and Prados, 2021), highlighting the need for mental healthcare to target depressive symptoms and the impact of prolonged life disruptions. The different trends of anxiety and depression also suggest the need for research to consider their unique etiology and developmental courses in addition to their comorbidity, especially when individuals experience chronic stressors.

More importantly, our findings indicate diverging trajectories of symptoms by parents’ mental health help-seeking. *Inconsistent seekers* and *non-seeking needers*, although differing in whether they had contacts with health or mental health professionals, were both characterized by unmet mental health needs and showed greater increases in depressive and anxiety symptoms than the other two groups. This highlights the importance of understanding the barriers preventing parents from seeking professional help. Based on a pre-pandemic review, worry about stigma, poor mental health literacy, and belief in self-reliance were the leading barriers to help-seeking among adults (Gulliver et al., 2010). In the current sample, stigma was less of a barrier, possibly due to the mental health advocacy and awareness campaigns (Cheng et al., 2016; Booth et al., 2018). However, belief in self-reliance was still a primary reason for not seeking help, and other factors (e.g., time constraints) might be specific to parents with increased childcare responsibilities leading to increased stress and less time to address their own needs. Paradoxically, pandemic-related stresses increase the need for mental health service while diminishing its availability. In addition to motivational and logistical barriers, access to in-person health and mental healthcare has been limited due to public health measures. Although policies and fundings have been devoted to promoting telehealth since the beginning of the pandemic, such transition, combined with heightened demands, is challenging both for providers and for individuals needing professional help (e.g., in the current sample, 20% of parents with unmet mental health needs indicated reluctance around telehealth as a factor). Our findings suggest that accessing professional mental health help is associated with lower risk for worsening symptoms among parents. This highlights the need to facilitate access to such services at all levels during and beyond the pandemic, including appropriate resource allocation at the policy level, change in public attitudes to service use, and reduction of physical access obstacles, e.g., continue to promote the use of technology-assisted consultation and intervention (Harris et al., 2020). Beyond the parents, it must be recognized that parenting is itself a “new frontier” (Johnson, 2020), with parental mental health problems likely disrupting child development in vulnerable families (Cluver et al., 2020). This magnifies the need for accessible mental health services for parents and, by implication, their children.

In addition to public policy and resources that increase access to mental healthcare, research has also identified a range of behavioral intervention strategies to promote help-seeking among adults. A meta-analysis found that improving mental health literacy and addressing mental illness stigma through psychoeducation or therapy can effectively promote seeking professional help for mental health concerns in the short term, and motivational enhancement strategies (e.g., resolving ambivalent attitudes toward treatment) show long-term effects (Xu et al., 2018). Additionally, some interventions target social support (e.g., promoting people’s awareness of their significant others’ or peers’ mental health well-being) and screening to promote mental health help-seeking (Hom et al., 2015). These strategies may help address the motivational (e.g., preference for self-reliance and stigma) barriers to mental health help-seeking identified in the current study. Notably, there is a lack of intervention strategies designed specifically for parents. Some programs engage parents to promote their mental health literacy; however, the goal is often to increase parents’ help-seeking for their children’s mental health problems, rather than attending to parents’ own mental health (Murphy et al., 2021; Peyton et al., 2022). Given that parents may face unique stressors and challenges in accessing mental health help, calibrating interventions to meet parents’ needs is an important next step.

This study has limitations that warrant caution in the generalization of findings and implications. First, this was not a representative sample. The final sample included mostly mothers and parents who were married or living with a common-law partner, and only a small proportion identified as ethnic minority. Over half of the sample held a college degree, a higher rate than the adult population in Ontario (Statistics Canada, 2017). The experience of stress and help-seeking in this sample may not generalize to other populations. For example, families that are more socioeconomically disadvantaged may face greater logistical barriers to accessing professional help. Furthermore, the findings may not generalize to other provinces and countries with different healthcare systems or sociocultural perspectives on mental health help-seeking. Despite this limitation, the findings still reveal important information on the relation between mental health help-seeking and trajectories of mental health symptoms and provide insights into potential barriers leading to unmet mental health needs. Second, the two waves of data were collected during peaks of COVID case surges and provincial lockdowns and likely captured parents’ depressive and anxiety symptoms at the most stressful times of the pandemic. They could not represent the full trajectories of parents’ mental health conditions across the past 2 years. Examining the ebbs and flows of individuals’ experiences across a variety of situational changes, especially the transitions between critical and non-critical times of the pandemic, would have important implications for understanding resilience and recovery from the stress. Third, several factors that may be associated with stress during the pandemic (e.g., anxiety symptoms, being an ethnic minority, and having financial difficulties) were related to longitudinal data attrition (i.e., not consenting or responding to follow-up; see Supplementary Table 1). Some parents at high risk for worsening symptoms and/or faced more challenges in help-seeking might not be captured by the results. Finally, this study measured parents’ help-seeking retrospectively, which may be associated with biases (e.g., parents experiencing worsening symptoms might tend to report unmet needs; parents might not accurately distinguish different types of mental health professionals). This study is intentionally descriptive, and we do not aim to make causal inferences on whether help-seeking leads to variations in mental health trajectories.

## Conclusion and Implications

This study constitutes one of the first investigations examining trajectories of parental mental health symptoms during the pandemic as related to mental health help-seeking, and provides evidence-based targets for promoting families’ well-being during and beyond the pandemic. Among parents with no perceived unmet mental health needs, anxiety symptoms decreased but depressive symptoms increased. Meanwhile, parents with perceived unmet mental health needs showed an alarming trend of increasing anxiety and greater increase in depressive symptoms. The findings suggest that COVID-19 has resulted in a mental health crisis, with those not consistently seeking professional help at the greatest risk for prolonged, elevated mental health difficulties. Currently, although some regions with effective monitoring of infection trends and high vaccination rates are slowly moving into a phase with reduced life disruptions, many regions still face continuing increases in the rates of COVID-19 infection, overwhelmed healthcare system, and significant disruptions to daily lives. More importantly, the pandemic has had profound economic, social, and health impact that leads to long-term stress and disadvantages for many families, even when the infection from COVID-19 itself is under control. Therefore, additional mental health resources are needed during and beyond this pandemic, as is awareness of the scope and salience of mental health challenges among parents. Services and policies should continue to promote help-seeking among parents, including devoting resources to publicly funded mental health service infrastructure, and targeting barriers to parents’ help-seeking.

## Data Availability Statement

The raw data supporting the conclusions of this article will be made available by the authors, without undue reservation.

## Ethics Statement

The studies involving human participants were reviewed and approved by the Hamilton Integrated Research Ethics Board (HiREB). The patients/participants provided their written informed consent to participate in this study.

## Author Contributions

XZ conceptualized the study, conducted analyses, and led the manuscript write up. AG and LA were involved in the design of the study and selection of measures. MJ and DJ were involved in data preparation and advising on analyses. All authors contributed to the article and approved the submitted version.

## Funding

The OPS project was supported by the Public Health Agency of Canada (#1819-HQ-000068).

## Conflict of Interest

The authors declare that the research was conducted in the absence of any commercial or financial relationships that could be construed as a potential conflict of interest.

## Publisher’s Note

All claims expressed in this article are solely those of the authors and do not necessarily represent those of their affiliated organizations, or those of the publisher, the editors and the reviewers. Any product that may be evaluated in this article, or claim that may be made by its manufacturer, is not guaranteed or endorsed by the publisher.

## Supplementary Material

The Supplementary Material for this article can be found online at: https://www.frontiersin.org/articles/10.3389/fpsyg. 2022.884591/full#supplementary-material
