# Supplementary material for: Mental Health Help-Seeking in Parents and Trajectories of Depressive and Anxiety Symptoms: Lessons Learned From the Ontario Parent Survey During the COVID-19 Pandemic
Source: Front Psychol. 2022 Jun 16;13:884591. doi: 10.3389/fpsyg.2022.884591 (PMC9243663; doi:10.3389/fpsyg.2022.884591)
Supplement: Supplementary file 1 [file Table_1.DOCX]

**Supplementary Materials**

**eTable 1.** Comparisons Between the Included Sample and Declined/Dropped-Out Sample^a^

|  | **Included sample**  **(n = 2439)** | **Declined/Dropped-out sample**  **(n = 4247)** | **Group comparison** |
| --- | --- | --- | --- |
| Age in years, mean (*SD*) | 39.47 (6.65) | 38.38 (6.92) | *t* (5243) = 6.33, *p* < .001 |
| Female, No. (%) | 2316 (95.0) | 3938 (92.9) | χ^2^(1) = 11.34, *p* < .001 |
| Ethnic minority^b^, No. (%) | 260 (10.9) | 534 (13.2) | χ^2^(1) = 7.31, *p* = .007 |
| Married, No. (%) | 2095 (86.0) | 3681 (87.0) | χ^2^(1) = 1.25, *p* = .26 |
| College degree, No. (%) | 1441 (59.3) | 2073 (49.2) | χ^2^(1) = 63.82, *p* < .001 |
| Working full-time, No. (%) | 1559 (64.0) | 2714 (64.3) | χ^2^(1) = 0.04, *p* = .84 |
| Have child(ren) < 5 years, No. (%) | 1264 (51.8) | 2260 (53.2) | χ^2^(1) = 1.17, *p* = .28 |
| COVID financial impact^c^, mean (*SD*) | 0.73 (0.90) | 0.81^d^ (0.93) | *t* (4926) = -3.27, *p* = .001 |
| Depressive symptoms |  |  |  |
| W1 CESD score, Mean (SD) | 11.52 (6.40) | 11.18^d^ (6.35) | *t* (5205) = 1.96, *p* = .05 |
| W1 CESD ≥ 10, No. (%) | 1410 (58.1) | 1849 (55.6) | χ^2^(1) = 3.51, *p* = .06 |
| Anxiety symptoms |  |  |  |
| W1 GAD score, Mean (SD) | 8.05 (5.76) | 8.27^d^ (6.13) | *t* (5396) = -1.40, *p* = .16 |
| W1 GAD ≥ 10, No. (%) | 845 (34.8) | 1225 (37.5) | χ^2^(1) = 4.49, *p* = .03 |

Note. CESD = the sum score on CES-D-10; GAD = the sum score on GAD-7.

^a^ This included declined (participated in W1 but declined to be contacted for follow-up; n = 3211) and dropped-out (consented to be contacted for follow-up but did not respond to W2; n = 1036) parents. All percentages were calculated using the non-missing sample size for each variable as the denominator.

^b^ Ethnicity status other than North American and European was coded as ethnic minority.

^c^ The impact of COVID on family financial obligations.

^d^ Among all variables in the table, only COVID financial impact, CESD, and GAD in the excluded sample had > 10% of missing data (missing rates = 32.2%, 21.6%, and 23.2% respectively).

**eTable 2.** Survey Questions on Mental Health Help-Seeking

| **Question** |
| --- |
| 1. Since the beginning of the COVID-19 pandemic have you seen or talked on the telephone (or virtually/online) to any of the following types of health-care providers because of concerns about your mental health? Please select all that apply:  a. Family doctor or general practitioner (yes/no)  b. Psychiatrist (yes/no)  c. Psychologist (yes/no)  d. Nurse (yes/no)  e. Social worker (yes/no)  f. Some other type of counsellor (yes/no)  g. None (yes/no) |
| 2. Since the beginning of COVID-19 pandemic, was there a time when you felt like you might need professional help with your own mental health, but you did not seek help? (yes/no)  If yes, why did you not seek help? Please select all that apply:  a. I thought I could manage it myself  b. I didnt know where to get help  c. I never got around to it (e.g., too busy)  d. It would have been too hard to schedule  e. I tried but the wait was too long  f. I didnt think professional help would do any good  g. It was going to cost too much  h. Getting to the place where they provide help was a problem  i. I was afraid of what others would think of me  j. Did not feel comfortable with virtual or telephone sessions  k. Other |

**eFigure 1**. Specification of the Structural Equation Model (Latent Change Score Model) Examining Change in Parents’ Depressive or Anxiety Symptoms from W1 to W2


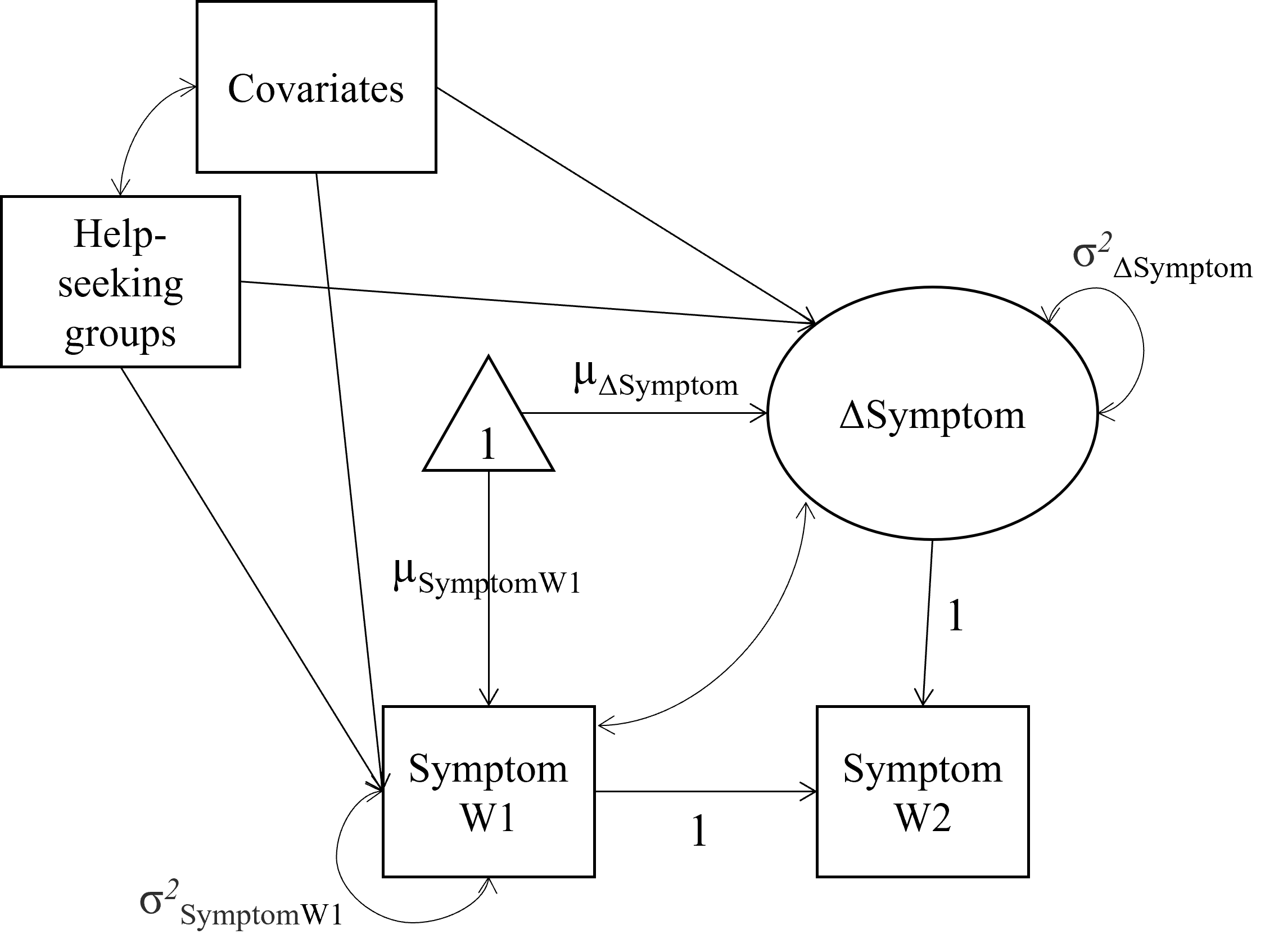


*Note*. Δ = Change from W1 to W2. In the full model, µ_SymptomW1_ and µ_ΔSymptom_ represent the level of mental health symptoms at W1 and the change between waves in the *no need* group. Mental health symptoms at W1 and ΔSymptom were regressed on all covariates. Correlations were specified between help-seeking groups and six covariates that differed significantly across groups (see Table 1; parent age, ethnicity, marital status, education, whether having child under 5, and COVID financial impact), and between symptoms at W1 and ΔSymptom. Correlations among covariates were pre-specified based on relevance and also added based on modification indices; parental age was correlated with parental gender, marital status, and whether having child(ren) under 5; whether having child(ren) under 5 was also correlated with parental marital status and education; parental education was correlated with marital status and employment; COVID financial impact was correlated with parental education, marital status, employment, and ethnicity.
